# Project RUSH: Implementing and evaluating a community-based teen pregnancy prevention program among Hispanic youth in rural South Texas

**DOI:** 10.1016/j.puhip.2026.100743

**Published:** 2026-02-06

**Authors:** Gerardo J. Pacheco, Howaida Werfelli, Ramalingam Shanmugam, Jose Betancourt, Alison Johnson

**Affiliations:** a School of Health Administration, Texas State University, San Marcos, TX, USA; b SALVERE, San Antonio, TX, USA; c Coastal Bend Wellness Foundation, Corpus Christi, TX, USA

**Keywords:** Teen pregnancy prevention program, Hispanic health, Sexual education

## Abstract

**Objectives:**

The burden of sexually transmitted infections, HIV, and unintended pregnancies is disproportionate among Hispanic youth (ages 15-19) in Texas as compared to other groups nationally. A community intervention, Project Realistic Understanding of Sexual Health (RUSH) was developed using the Making Proud Choices, an evidence-based teen pregnancy program (TPP). This study aimed to present the use of a community-based participatory research (CBPR) framework in designing and implementing a sexual health intervention for rural Hispanic youth and participant outcomes (knowledge, attitudes, and behaviors).

**Study design:**

Single group Pre/Post Design employing a CPBR approach.

**Methods:**

A CBPR approach and programmatic evaluation were utilized to design and deliver an evidence-based teen pregnancy prevention program (TPP). A pre-post questionnaire was self-administered to eligible and consenting participants who were recruited via convenience sampling. Project RUSH was delivered over 8-week sessions in community centers throughout the 5-county service area.

**Results:**

In total, 160 youth were recruited, and 112 participants completed the intervention with the pre-and-post assessments. The majority self-identified as Hispanic/Latino (76%). Increased knowledge of pregnancy, sexually transmitted infections (STIs), and HIV/AIDS were statistically significant after the intervention. Respondents’ feedback demonstrated satisfaction in the program (setting, facilitators, curriculum).

**Conclusion:**

Input and engagement from community stakeholders helped refine the implementation of RUSH. Although a group change was observed, additional research, community engagement, and further analyses are needed to assess long term and support positive sexual behaviors among the youth in rural South Texas.

**Implications and contributions:**

The findings support the continued need to develop tailored sexual education community interventions for youth in rural communities.

## 1 Introduction

### 1.1 Sexual and reproductive health within a global context

Sexual and reproductive health (SRH) among adolescents remains a primary global public health concern [1,2]. The World Health Organization (WHO) defines sexual health as “a state of physical, emotional, mental, and social wellbeing in relation to sexuality” and was identified target Sustainable Development Goal (SDG 3.7) in 2015 to ensure universal access to sexual and reproductive health-care services [3]. Adolescents aged 15-19 are the most vulnerable to teen pregnancy [2], sexually transmitted infections (STIs) [4], multiple partners [5], and other risks associated with sexual activity [6]. Annually, an estimated 21 million females aged 15 to 19 living in developing regions become pregnant, with an estimated 12 million resulting in live births [7]. According to United Nations 2024 modeling data, the median fertility rates (i.e., the number of live births in a given year per 1000 women) in 15 year-olds varied by global region: 28.6 births in Africa; 12.2 in Latin America and the Caribbean; 3.4 in Asia; 1.3 in Europe; and 1.2 in North America [8]. Similarly, among 16 year-olds the projected median rates ranged from: 54.2 births per 1000 in Africa; 23.7 in Latin America (and the Caribbean); 8.0 in Asia; 3.1 in Europe; and 3.6 in North America [8]. The fertility rate among 17-year-olds was highest in Africa and Latin America (84.8 per 1000 and 37.5 per 1,000, respectively) [8].

Sexually transmitted infections (STIs), caused by viruses, fungi, bacteria, or protozoa, remain a public health concern. Despite available screening and treatment, the burden of STIs remains higher among adolescents [[9], [10], [11], [12]]. Adolescence encompasses pivotal milestones in cognitive and physical development [9] that help shape the individual's behaviors respective to sexual health [4,11,13]. Though global research in SRH in younger adolescents is limited [1,9], a recent narrative review identified chlamydia as the most occurring global STI: 129 million new cases in persons 15-49 with an incidence rate of 29 per 1000 males and 36 per 1000 females [12].

Adolescents in low-and-middle income countries (LMIC) face additional challenges and barriers regarding SHR. Half of an estimated 21 million annual pregnancies that occur among adolescent females (ages 15 to 19) in LMIC are unintended [7]. In LMIC, 14 million adolescent women have an unmet need for modern contraception-defined as those wishing to avoid pregnancy while not currently employing modern methods [7]. While recent research [14] identifies adolescent salient predictors of risk behaviors (e.g., self-regulation, care giver control, substance abuse, and sexting) within a broad global context, emerging [4,13,15] literature on adolescents in LMIC or rural settings expand into the nuanced environmental, social, interpersonal, and individual-level challenges (e.g., limited knowledge/education, gender norms, healthcare system/access to care and services). Global policies outline the need for systems-level approaches [15] to prioritize SHR research [9,16] and address the needs of underserved communities in rural and/or low- and middle-income countries [4,7].

### 1.2 SHR trends among US adolescents

Despite recent national declines in teen birth rates, adolescents in the United States continue to experience significantly higher rates of unintended pregnancies and STIs [11,17] compared to their peers in other high-income nations. In 2018, the overall United States (U.S.) birth rate was 17.4 per 1000 for females aged 15-19 [18]. Between 2016 and 2021, while the overall rate in females aged 15-19 in the U.S. dropped nearly 7.0% (13.9 per 1000), the birth rate was 20.3 per 1000 for 2021 among adolescents in Texas [19]. The birth rate for all females aged 15-19 was still higher in Texas than in most of the other states (25.3 per 1000) for 2018 [18]. Hispanic youth (females 15-19) were disproportionately impacted (34.4 per 1000) for 2018 [18].

One in every four US sexually active adolescents (ages 15-24) are estimated to be living with an STI [11]. Adolescents (ages 15 to 24) made up nearly half of the 26 million new STIs for 2018 [20]. According to 2021 Centers for Disease Control and Prevention (CDC) surveillance data, nearly half of the reported U.S. cases of syphilis, gonorrhea, and chlamydia were in adolescents and young adults (ages 15-24) [21]. More specifically, adolescents and young adults (AYA) accounted for 57.7% of all chlamydia cases reported in the U.S. [21]. Females 15-19 shared the second highest burden of new cases behind the 20-24 age group [21]. The data does not consider other demographic characteristics or risk factors that may further exacerbate sexual health behaviors among AYA. The disparity is further widened, for example, when examining sexual behaviors among sexual and gender minority youth (SGMY) [22].

According to Youth Risk Behavior Surveillance System (YRBSS) data, nearly 1 in 3 U S. high schoolers reported to have ever had sexual intercourse [23]. In the U.S., 3.2% indicated that they had sexual intercourse for the first time before the age of 13, compared to 4.7% in Texas [23]. Other high-risk sexual behaviors were consistently reported among the sampled U.S. high schoolers when compared to those in Texas. However, 20.5% in Texas indicated that they did not use any contraceptive method to prevent pregnancy during last sexual intercourse with an opposite-sex partner as compared to 13.7% for all U.S. youth [23].

In a 2004 study, 89% of sampled 14- to 19-year-old Mexican Americans living in rural South Texas were sexually active [24]. In the proposed 5 county service delivery area, 4.5% of all live births were among females aged 15-19 in 2016. The teen birth rate among the target population in the 5 counties ranged from 42.7 to 58.8 [25]. Texas had the second-highest rate of repeat teen births in the country in 2019 [26]. In 2019, 18% of births to Texas teens aged 15-19 were repeat births. Additionally, among unmarried young women aged 20-24, more than half of births were repeat birth [27].

### 1.3 The Coastal Bend Wellness Foundation

The Coastal Bend Wellness Foundation (CBWF) is based in Corpus Christi, Texas. The Corpus Christi Health Service Delivery Area (HSDA) covers 11,000 square miles and 12 counties including Aransas, Bee, Duval, Jim Wells, Kennedy, Kleberg, Live Oak, McMullen, Nueces, Refugio, and San Patricio [28]. The CBWF received a grant from the U.S. Office of Adolescent Health (AOH) to mitigate the problem of high-risk behaviors among the youth of rural counties in the Corpus Christi (i.e., serving the communities along Coastal Bend region) area, thus aligning with the federal mandate to reduce pregnancies in adolescents [29] utilizing evidence-based teen pregnancy prevention (TPP) initiatives [30]. CBWF is the only HIV healthcare provider in the Coastal Bend region.

### 1.4 Making Proud Choices!

The Making Proud Choices! (MPC) curriculum has been implemented in other communities in the US [31,32]. CBWF selected MPC the evidence-based curriculum to implement in this target population based on evidence base guidelines, curriculum costs, and feasibility of adopting the TPP to Hispanic adolescents in the Coastal Bend region. The U.S. Department of Health and Human Services Office of Population Affairs (OPA) supports the use of MPC as an evidence-based TPP [33,34]. The effectiveness has been recently validated in a cluster randomized trial among 9th and 10th graders in 4 U S. cities [31].

### 1.5 Project RUSH

However, to date, MPC has not been evaluated among Hispanic youth in a rural setting. This gap is significant because previous efficacy trials for MPC have primarily focused on African American youth in urban centers. Furthermore, rural Hispanic adolescents face unique barriers to sexual health access, including geographic isolation and specific cultural norms, which necessitates a tailored evaluation of evidence-based interventions in this specific context. This is the first study to our knowledge that utilized the MPC curriculum among Hispanic youth in a rural setting.

Project RUSH (Realistic Understanding of Sexual Health) was an innovative community intervention to educate high school youth and their families in the creation of an environment that encourages healthy adolescence and addresses sexual risks in healthy decision-making and TPP. Public health researchers collaborated with CBWF staff and leadership to design, implement, and evaluate Project RUSH after conducting a community needs assessment in 2018. Between 2022 and 2023, Project RUSH was delivered to 254 individuals throughout five counties in South Texas: Aransas, Bee, Kleberg, Nueces, and San Patricio. These counties demonstrate an unmet need for healthcare services including access to SRH resources and education. Project RUSH aligns with the WHO's SDG 3.7 by increasing knowledge on sexual behavior among adolescents through an evidence-based curriculum.

While the overarching goal of Project RUSH was to reduce the burden of unintended pregnancies and STI rates among Hispanic youth through long-term behavioral change, the purpose of this manuscript is to describe the community-based participatory research (CBPR) approach in designing, implementing, and evaluating a TPP in a rural community of predominant Hispanic adolescents and present descriptive outcomes for the intervention (e.g., participants’ sexual-related attitudes, beliefs, and behaviors).

## 2 Methods

### 2.1 Planning and community engagement

A community needs assessment was conducted in 2018 in four of the five service counties. The community needs assessment summarized the health profile for each county. Data was reported on youth birth rates, pregnancy rates among the youth, prevalence of STIs (including HIV), sexual risk behaviors, and existing disparities. Other information on community resources, stakeholders, social determinants of health, and co-occurring risk behaviors that impact teen pregnancy, STIs, and sexual risk-taking (i.e., binge drinking and alcohol use) rates among the target population was identified and mapped. A pilot study was conducted in 2020 (from October to December). The logic model for Project RUSH describes how the program was designed for community implementation throughout the 5-county service area (Aransas, Bee, Kleberg, Nueces, and San Patricio) (Supplement Fig. 1). Out of the 19 community stakeholders, only four officially committed to support Project RUSH.

### 2.2 Design

Formative, process, and outcome evaluations were conducted during the implementation of Project RUSH. The formative evaluation was conducted in the first year of program implementation and focused on establishing project merit, maintaining fidelity to the program and adaptation of guidelines, and ensuring feasibility and appropriateness to the target population. The process evaluation was performed for the duration of the project to monitor activities, as outlined by the logic model. Finally, the programming goals for each year were assessed for the outcome evaluation. The pilot study afforded the project the opportunity to implement the program in a real setting with fully trained facilitators and to make modifications to the approach and delivery. These included the potential need to utilize incentives to help engage the participants throughout the life of the project. The study design is an observational cross-sectional with matched repeated measures (i.e., pre, and post-assessment).

### 2.3 Recruitment and data collection

CBWF recruited eligible participants during health fairs and community events. Convenience sampling was used to recruit eligible participants (i.e., youth aged 15-19 residing in the five-county service area). CBWF adhered to the MPC curriculum resources (i.e., training materials, lecture materials/lessons, and data collection forms).

### 2.4 MPC content and tools

The MPC curriculum consists of 60-min 8-week sessions that address the youth's knowledge of HIV/AIDS and STIs, unintended pregnancy and its outcomes, condom use, and negotiation skills [34]. Additionally, the youth's attitudes on condom usage, safer sex, and contraception are discussed. Perceptions of risk and skills/self-efficacy are part of the MPC curriculum. The educational intervention consisted of multi-site (i.e., community centers), and multi-sessions, between January 2022 through June 2023 following the recommended format (e.g., 8-week sessions). A self-administered pre-assessment was completed at the start of session 1. The extensive questionnaire included 5 sections (with over 120 items), with the first section focused on collecting participant's demographic information (age, gender, race/ethnicity including asking participants to self-report on Hispanic status). Participants were asked to rate Likert-scaled statements on sexual attitudes (i.e., 5-point agreement/disagreement items) as part of the second section (e.g., “How would the following people feel about having sex in the next 3 months”) for themselves, “most people who are important [to them]”, sexual partner, parents (individually), and friends. Refer to Appendix 1 for the summarized statements included as repeated measures (pre/post assessment) for the second section.

The third section required respondents to rate the level of ease for 3 questions: 1) *How easy or hard would it be for you to not have sex in the next* 3 months*?* 2) *How easy or hard would it be for you to get your partner to use condoms during sex, even if they didn't want to?* and 3) *How easy or hard would it be to use condoms when you have sex?* The fourth section was a self-assessment on participant's sexual behaviors (e.g., *the first time you had sexual intercourse, did you use a condom?*) In addition to the binary response options, participants were given an option to select “I have never had sexual intercourse.” Another set of items within this fourth section specified the occurrence for specified behavior (e.g., wearing a condom) over the past 3 months. There were 17 items in total for this section.

Knowledge on STIs was assessed in the fifth section using 24 True/False statements. The sixth section included 13 additional True/False items on “Personal attitudes” (e.g*., A few times, I have given up doing something because I thought too little of my ability*). A post-assessment was completed at the end of session 8. Except for the first section (i.e., the demographics), the items were repeated on both assessments. Additionally, the post-assessment questionnaire elicited participant feedback at the end. These tools were existing components of the MPC curriculum delivery (i.e., no modifications were done in the data collection instruments) in order to adhere to the fidelity of the TPP.

### 2.5 Data analysis

Data management and statistical analyses were performed using Microsoft Excel and Stata 14. Charts were created using Excel. Descriptive statistics, including frequencies, percentages, means, and standard deviations, were calculated to summarize participant demographics and baseline characteristics. Participants who completed only the pre-assessment were excluded from the final effectiveness analysis. To evaluate the intervention's impact, individual knowledge items were dichotomized into binary outcomes (correct vs. incorrect). The significance of changes in the proportion of correct responses between pre- and post-assessment was evaluated using McNemar's test for paired nominal data (i.e., the knowledge items). Attitudinal items were tabulated as nominal responses (level of agreement) to assess shifts in participant perspectives. Changes in attitudes from pre-to post-assessment are presented descriptively to illustrate trends in agreement, rather than evaluated for statistical significance.

### 2.6 Ethical considerations

The study protocol was reviewed and approved by the Salus IRB Review Board. Prior to data collection, the research team provided a comprehensive explanation of the study's purpose, procedures, potential risks, and benefits to all prospective participants and their guardians. Parents or guardians unable or unwilling to provide written consent to their child to participate in MPC were excluded from the study (e.g., not able to participate in the educational sessions). The written consent forms were securely stored by trained CBWF staff. Surveys and forms were de-identified to remove names or personal identifiers.

## 3 Results

Overall, 112 participants completed both the pre- and post-assessments. The majority were recruited during the second year of program implementation. Forty-six participants completed only the pre-assessment and were excluded from the analyses. The sample characteristics are shown in Table 1. Less than half our sample was female and less than 43% male. Nearly 76% self-identified as Hispanic/Latino. Only two participants reported as having had children. The average age was 16.2 with a range of 13 to 20.

**Table 1:** Demographics for participants.

Variable	Pre-Assessment^a^ Participants, N = 46 (%)	Pre and Post^b^ Participants, N = 112 (%)
Year of Participation		
Year 1 (2022)	8 (17.4)	30 (26.8)
Year 2 (2023)	38 (82.6)	82 (73.2)
Age		
Mean	16.2	16.2
Median	16.0	16.0
Range	[13, 20]	[13, 26]
Standard Deviation	1.5	1.7
Gender		
Male	22 (47.8)	48 (42.9)
Female	22 (47.8)	55 (49.1)
Transgender	1 (2.2)	6 (5.4)
Does not Identify/Other	1 (2.2)	3 (2.68)
Race		
White only	16 (34.8)	48 (42.9)
Two or More Races	14 (30.4)	36 (32.1)
Black/African American only	4 (8.7)	7 (6.3)
American Indian/Alaska only	3 (6.5)	8 (7.1)
Native Hawaiian/Pacific Islander only	1 (2.2)	-
No Response	8 (17.4)	13 (11.6)
Hispanic/Latino		
Yes	41 (89.1)	88 (78.6)
No	5 (10.9)	16 (14.3)
No Response	-	7 (6.3)
Had Children (*Indicated #)*		
Yes (at least 1)	5 (10.9)	2 (1.8)
None	41 (89.1)	110 (98.2)
County of Residence		
Nueces	30 (66.7)	80 (71.4)
San Patricio	4 (8.9)	5 (4.5)
Kleberg	1 (2.2)	4 (3.6)
Bee	-	3 (2.7)
Aransas	-	4 (3.6)
County not specified	10 (22.2)	16 (14.3)

a Only includes participants who completed the Demographics and Pre-Assessment.

b From Participants that completed both the Demographics, Pre-Assessment, and Post-Assessment, Pre-assessment includes participants that have not completed the post-assessments.

### 3.1 Attitudes

The responses on attitudes regarding sexual practices were collapsed into three categories to illustrate positive agreement, a neutral stance, or disagreement (See Supplementary figures). Overall, a reduction in negative (disagreement) attitudes was observed in the post-assessment across the four presented items: the likelihood of sexual intercourse in the next 3 months; agreement regarding sexual intercourse resulting in HIV; agreement regarding sexual intercourse resulting in STIs; and agreement regarding sexual intercourse resulting in pregnancy.

**Fig. 1:**
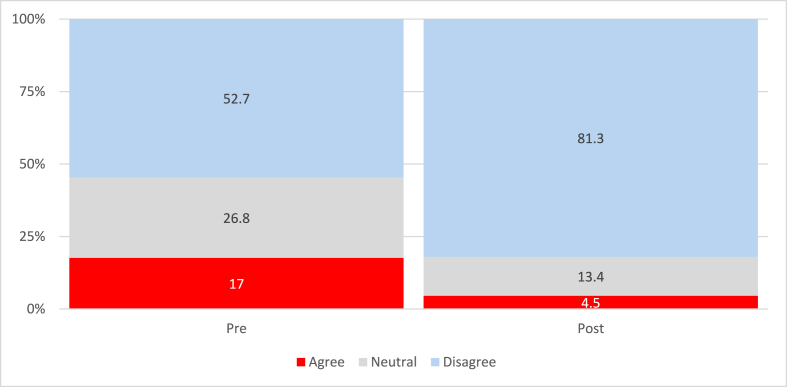
Condoms Cost too Much.

An increase in the proportion of participants who agree with the statements (i.e., protective behaviors) was noted. Conversely, fewer respondents disagreed with the post-assessment. There was a reduction in agreement on the likelihood of using condoms in the next 3 months after completing the education session. Less than 10% of participants in the pre-assessment accounted for a non-response for this item as compared to 25% in the post-assessment (Supplementary Fig. 6). Regarding condom accessibility, the biggest gain post-intervention was regarding the attitude that condoms cost too much from 53% to 81% disagreeing with the statement (Fig. 1).

### 3.2 Knowledge

The changes in the postal assessment were statistically significant in the 12 knowledge items presented (Table 2). Participant knowledge improved after completion of the intervention. The highest increase (50%) was reported for correctly recognizing that HIV transmission does not occur from sharing a sink, shower, or toilet seat with someone living with HIV/AIDS (item #12). The baseline assessment score for this item was the lowest (24%). The highest baseline mean score (71%) was for item #9 (“a person can have HIV/AIDS and give it to other people even if the person does not look sick”). The lowest mean score (31%) after participating in the program was reported for discharge from penis as a symptom of an STI. Participants scored the lowest increase (12%) after the program for items #8 (a person can have HIV/AIDS and give it to other people even if the person does not look sick) and #9.

**Table 2:** Selected knowledge items.

Item	Mean Pre	Mean Post	Mean Post -Pre	SD Pre	SD Post	SD Post-Pre	McNemar	*p*-value
1. Discharge from penis	0.31	0.60	0.29	0.47	0.49	0.02	20.48	<0.001*
2. Burning when urinating	0.65	0.85	0.20	0.48	0.36	−0.12	14.24	<0.001*
3. Sore as a symptom	0.55	0.78	0.23	0.50	0.42	−0.08	16.03	<0.001*
4. A common symptom is discharge from the vagina	0.57	0.85	0.28	0.50	0.36	−0.14	25.97	<0.001*
5. Feeling healthy indicates no STI	0.58	0.84	0.26	0.50	0.37	−0.13	22.73	<0.001*
6. Women w/STIs can get infections in their tubes/uterus	0.56	0.75	0.19	0.50	0.43	−0.07	14.08	<0.001*
7. A mother can transmit STI to a baby	0.43	0.82	0.39	0.50	0.38	−0.12	38.72	<0.001
8. If a person has an STI, then the sexual partner can get	0.65	0.77	0.12	0.48	0.42	−0.06	5.45	0.0196*
9. A person can have HIV/AIDS and give it to other people even if the person does not look sick	0.71	0.83	0.12	0.45	0.38	−0.07	6.26	0.0124*
10. HIV is present in blood, semen, and vaginal fluid	0.56	0.86	0.30	0.50	0.35	−0.15	26.56	<0.001*
11. A girl cannot get pregnant the first time she has sex	0.66	0.88	0.22	0.48	0.33	−0.15	16.00	<0.001*
12. HIV/AIDS can be transmitted by sharing a sink, shower, or toilet seat with someone who has HIV/AIDS	0.24	0.74	0.50	0.43	0.44	0.01	47.52	<0.001*

*Statistically significant at a value of < 0.05.

### 3.3 Feedback

Three key themes emerged after reviewing the post-assessment feedback: 1) the program was interactive/fun; 2) the program improved their knowledge/was an opportunity to learn; and 3) satisfaction with the program setting/facilitators. For example, for the first theme, participants indicated specific activities that helped them “learn about … [what] we talked about.” Examples of learning “more things,” “… about STI's,” “… about sex,” “[about] ways to stay safe from STDS/HIV and unplanned pregnancies,” and “about something I always had questions about” reflected participants' comments on their knowledge. Finally, regarding the setting and facilitators, participants commented on “How safe and non-judgmental it was,” or how “the teacher, it was a comfortable setting.”

## 4 Discussion

Overall, there was a reduction in perceived “negative” attitudes (i.e., disagreement with protective norms) and an increase in participants agreeing with statements regarding the risks of sexual intercourse resulting in HIV, STIs, and pregnancy. Our findings suggest that knowledge related to SRH increased after participating in our multi-session TPPP, as illustrated by Table 2 in the increased proportion of participants correctly identifying STI symptoms and the risks of unprotected sexual practices. Contrary to expected outcomes, there was a reduction in agreement regarding the “likelihood of using condoms in the next 3 months” after the education session. However, this finding is notable for its data limitations: non-responses for this specific item jumped from less than 10% in the pre-assessment to 25% in the post-assessment, suggesting survey fatigue or discomfort may have skewed this specific data point. Our TPPP, Project RUSH, successfully recruited and retained the specific understudied population identified in the study aims: 76% of the 112 participants who completed the intervention self-identified as Hispanic/Latino, validating the program's reach within the rural target community.

### 4.1 Risks and access to barriers

While participants demonstrated high self-efficacy in negotiating condom use, they identified cost—rather than confidentiality—as the primary barrier to access. This finding invites a broader interpretation through the Socio-Ecological Model. In a recent systematic review of adolescent sexual behavior, the authors noted that individual knowledge is insufficient when structural determinants (such as economic constraints and lack of youth-friendly services) remain unaddressed [13]. Our findings mirror the “interconnected determinants” described by Maguena et al. (2025): distinct barriers exist at the individual level (ambivalence), the interpersonal level (communication gaps), and the structural level (cost) [13]. Where Barral et al. (2020) [35] highlight confidentiality as the dominant rural barrier, our participants’ focus on cost suggests that the CBPR approach may have successfully mitigated social stigma (the interpersonal barrier), thereby revealing the underlying economic (structural) barrier. This aligns with the conclusion from Koumba Maguena et al. highlighting the need for multi-sectoral interventions that extend beyond the biology of sex and include the environment in which adolescents access protection [35].

### 4.2 The role of safe environments

In a 2016 report, 58.3% of Texas public schools teach abstinence-only curriculum, 16.6% of Texas public schools teach abstinence-plus curriculum, and 25.1% of Texas public schools provide no sexual education [36]. Unsurprisingly, delivering the Project RUSH sessions within our rural 5-county service area in school settings was not feasible due to community resistance against SHR education. Although the educational sessions were conducted in community centers, participants felt the facilitators and setting provided a safe environment to discuss and learn about SRH. This aligns with the work from Wilkins et al. (2022) who argue that effective school-based health interventions must go beyond curriculum to establish “Safe and Supportive Environments” (SSE) [17]. By utilizing a CBPR approach with trusted local facilitators, Project RUSH effectively operationalized the SSE component of the Wilkins model. The data suggests that this supportive climate successfully mitigated the “interpersonal” barrier of stigma (confidentiality), which Barral et al. (2020) identify as the primary obstacle in rural Hispanic communities [35]. Because the environment was perceived as safe, participants were able to look past the fear of judgment and identify the practical impediments to their health.

### 4.3 Improvements in knowledge

Participation in the program resulted in statistically significant increases in knowledge regarding STIs and contraceptive methods. This aligns with outcomes from the original *Making* MPC randomized control trials [31] and reinforces the necessity of evidence-based TPP curriculums for Hispanic youth. However, beyond knowledge acquisition, our findings regarding attitude shifts and access barriers offer distinct insights when viewed through the lens of recent rural health literature.

A notable finding was the post-intervention increase in “neutral” responses regarding sexual health attitudes. Rather than interpreting this as a failure to persuade, we suggest this reflects a state of cognitive “ambivalence” common among rural Latino youth. Barral et al. (2020) identified that rural Latino adolescents often struggle with conflicting values—balancing traditional cultural expectations (*familismo*, sexual taboos) with new sexual health information [35]. In this context, the shift from “negative” beliefs to “neutrality” likely represents an “unfreezing” of prior misconceptions and a transitional stage of critical thinking, consistent with the ambivalence regarding pregnancy intentions observed in similar rural populations [35].

### 4.4 TPPP using CBPR

The successful implementation and retention rates in this study underscore the value of the CBPR approach. Recent work from Champion et al. [37] emphasizes that “one-size-fits-all” interventions often fail in rural Mexican American settings due to the complex interplay of poverty, culture, and limited mental health resources. Our study supports Champion's conclusion that developing “culturally appropriate interventions tailored to the target population” is a prerequisite for feasibility [37]. By utilizing trusted community partners to navigate these rural stressors, Project RUSH demonstrated that complex TPP interventions are viable in these hard-to-reach service areas.

Our findings underscore that implementing TPP programs in rural Hispanic communities requires more than just cultural translation; it requires a workforce strategy that addresses geographic isolation. While the CBPR approach successfully engaged local stakeholders, the sustainability of such interventions hinges on the ongoing quality of these community facilitators. Sontag-Padilla et al. (2023) emphasize that positive behavioral outcomes in programs like MPC! are strictly dependent on implementation fidelity [38]. However, they note that traditional in-person training is often cost-prohibitive for smaller organizations, presenting a significant barrier for rural sites where travel costs and staff coverage are limited.

This has direct implications for the scalability of Project RUSH. In our study, reliance on local partners fostered trust, but maintaining high fidelity across a dispersed 5-county service area remains a logistical challenge. Future iterations of this intervention should consider the “online simulator-based training” models proposed by Sontag-Padilla et al. (2023) [38]. Their research indicates that simulation-based training can be non-inferior to in-person training for creating safe, inclusive environments—a critical skill when addressing sensitive topics with Hispanic youth. Adopting such accessible training modalities would allow rural programs to maintain the high fidelity required for effectiveness without the prohibitive “travel and substitute” costs that often stifle rural health initiatives.

Though a change was observed (collectively) for educating the youth, additional research, community engagement, and further analyses are needed to assess long term and sustained changes in behavior and how that might translate to pregnancy and STI's among the youth in rural South Texas.

#### 4.4.1 Strengths and limitations

This is the first study to our knowledge that utilized the MPC curriculum among Hispanic youth in a rural setting. We posit that the curriculum can be further tailored to meet the needs of this specific target population and minimize the threats to internal and external validity. The modality of the pre-post questionnaires in our implementation of MPC is a factor contributing to the internal consistency of the measures. The pre- and post-self-administered questionnaires contained 143 and 123 items, respectively, both as paper-based submissions. It is our opinion that this may contribute to response bias. Particularly, we observed unexpected results for the participants' likelihood of engaging in sexual intercourse (See Supplementary Fig. 2) in which a larger proportion of participants *before* the intervention disagreed with the statement (37%) as compared to 19% in the post assessment. Moreover, nearly 27% in the post-assessment provided no response compared to 11% during the initial session. This might suggest participants' survey fatigue towards the conclusion of the intervention. Further research will explore the internal consistency of repeated measures and account for the target population's preferences in employing technology to improve/enrich learning and complete the assessments.

## Ethical statement

The study protocol was reviewed and approved by the Salus IRB Review Board.

## Statements of authorship

A.J. and H.W. conceived and carried out the research (program planning and implementation). G.J.P. worked on the evaluation and statistical analyses, performed computations, and wrote the manuscript. R.S. and J.B. interpreted results and worked on the manuscript. All authors discussed the results and contributed to the final manuscript.

## Declaration of generative AI and AI-assisted technologies in the manuscript preparation process

During the preparation of this work the authors used Google Gemini in revising original written content and improving the organization of sections. After using this tool/service, the authors reviewed and edited the content as needed and take full responsibility for the content of the published article.

## Funding

This research was supported in part by funding by the US Office of Adolescent 10.13039/100018696Health, Award #: TP1AH000267.

## Declaration of competing interest

The authors have no conflict of interest, financial or otherwise.
