# Supplementary material for: Project RUSH: Implementing and evaluating a community-based teen pregnancy prevention program among Hispanic youth in rural South Texas
Source: Public Health Pract (Oxf). 2026 Feb 6;11:100743. doi: 10.1016/j.puhip.2026.100743 (PMC12914447; doi:10.1016/j.puhip.2026.100743)
Supplement: Multimedia component 3 [file mmc3.docx]

Supplemental Figure 3: Agreement that Engaging in Sexual Intercourse Results in HIV
