# Supplementary material for: Project RUSH: Implementing and evaluating a community-based teen pregnancy prevention program among Hispanic youth in rural South Texas
Source: Public Health Pract (Oxf). 2026 Feb 6;11:100743. doi: 10.1016/j.puhip.2026.100743 (PMC12914447; doi:10.1016/j.puhip.2026.100743)
Supplement: Multimedia component 7 [file mmc7.docx]

**Appendix 1: Making Proud Choices! Curriculum Pre/ Post Assessment Sample of Items related to Sexual Attitudes (Section B)**

Approving behavior (1 through 5 Likert-scaled level of agreement)

- If I have sex, then I will be more popular with boys.
- If I have sex, then I will be more popular with girls
- If I have sex, I will get a bad reputation.
- If I have sex, I will get HIV.
- If I have sex, I will get a sexually transmitted disease (STD).
- If I have sex during my teen years, pregnancy could occur.
- If I have sex during my teen years, then my parents will find out.
- If I have sex, and my parents find out, then they will be angry at me.
- If I have sex before I am married, then God is likely to be angry at me.
- If I have sex during my teen years, then I am less likely to graduate from high school.
- If I have sex during my teen years, then I am less likely to have the career that I am hoping for.
- I plan to have sex in the next 3 months.

Items related to not having sex. *How much do you agree or disagree with each of the following statements about you not having sex?* (Reversed 1 through 5 Likert-scaled level of agreement).

- If I do not have sex, people will call me names.
- If I do not have sex, no one will want to go out with me.
- If I do not have sex with my partner, then they will break up with me.
- If I do not have sex my parents will be proud of me.
- If I do not have sex during my teenage years, I will be proud of myself.
- Not having sex will help me further my education.
- Not having sex will help me focus on getting a good job.
- I will not have sex in the next 3 months.

Statements about condoms.

- Condoms help prevent pregnancy.
- Condoms help prevent STDs.
- Condoms help prevent HIV.
- A lot of times condoms break when you are using them.
- When a condom is used, sex still feels good.
- When a condom is used, sex is more fun.
- If I used a condom, sex would not feel as good.
- Sex feels unnatural when a condom is used.
- Condoms are embarrassing to use.
- Condoms make you not want to have sex because you have to stop to put one on.

Items related to the level of disagreement for sexual partner’s attitudes on condoms. (Reversed 1 through 5 Likert-scaled level of agreement).

- Saying we have to use a condom would make my sexual partner think I am having sex with other people.
- Saying we have to use a condom is like saying to my sexual partner, “I don’t trust you.”
- My sexual partner is likely to break up with me if I said we had to use a condom.
- If I had a condom with me, my sexual partner would not like it.
- My sexual partner would be happier if we used a condom.
- Condoms cost too much.
- It is hard for me to get condoms.
- It is too much trouble to carry around condoms.
- I can get condoms.
- It is easy for me to have a condom with me all of the time.
- I can get my sexual partner to agree to use a condom, even if they don't want to.
- I can say to my sexual partner that we should use a condom.
- Before we are ready to have sex,
- I can talk to my sexual partner about using a condom.
- I can put a condom on without turning my sexual partner off.
- I cannot talk to my sexual partner about using condoms.
- If I am sexually aroused, I can stop before sex to use a condom.
- I can say no to sex if my sexual partner and I do not have a condom.
- I can stop sex to get a condom, if I do not have one.
- I can use a condom, even if the room is dark.
- I can get my sexual partner to agree to use a condom without turning them off.
- I am sure that I can use a condom if I have sex.
- I will try to get my sexual partner to agree to use condoms if we have sex in the next 3 months.
- I plan to use condoms if I have sex in the next 3 months.
